# Measuring the Subjective Signal Strength: Validating Persian Vividness of Visual Mental Imagery Questionnaire‐2

**DOI:** 10.1002/brb3.71203

**Published:** 2026-01-15

**Authors:** Mohammad Atashrooz, Fatemeh Mirzai, Maede Amin Roaya, Hannaneh Fayyaz Rouhi, Arash Ghadir, Hoda Doosalivand, Amir Sam Kianimoghadam

**Affiliations:** ^1^ Student Research Committee, Faculty of Medicine Shahid Beheshti University of Medical Sciences Tehran Iran; ^2^ Department of Clinical Psychology, School of Medicine Shahid Beheshti University of Medical Sciences Tehran Iran; ^3^ Department of Psychology, Faculty of Economics and Social Sciences Bu‐Ali Sina University Hamedan Iran; ^4^ Department of Health Psychology, School of Behavioral Sciences and Mental Health (Tehran Institute of Psychiatry) Iran University of Medical Sciences Tehran Iran; ^5^ Department of Psychology, Faculty of Humanities Islamic Azad University of Lahijan Lahijan Iran; ^6^ Department of Psychology, Faculty of Humanities Islamic Azad University of Amol, Ayatollah Amoli Branch Amol Iran

**Keywords:** imagination, mental imagery, Persian, psychometrics, reality monitoring, Vividness of Visual Imagery Questionnaire, VVIQ

## Abstract

**Introduction:**

Although visual mental imagery has been widely researched, a lack of valid measures in Persian‐speaking populations has limited cross‐cultural, developmental, and clinical research on imagery vividness and its role in reality monitoring.

**Methods:**

We translated, culturally adapted, and psychometrically validated the Persian version of the Vividness of Visual Imagery Questionnaire‐2 (VVIQ‐Pr2) in this cross‐sectional study. Our sample was 630 Persian speakers. Participants completed the VVIQ‐Pr2 together with the Vividness of Motor Imagery Questionnaire (VMIQ‐2), the Spontaneous Use of Imagery Scale (SUIS), the Generalized Anxiety Disorder‐7 (GAD‐7), and the Ten Item Personality Inventory (TIPI). Confirmatory factor analysis and multi‐group modeling were also conducted.

**Results:**

Confirmatory factor analysis supported a unidimensional structure with correlated residuals demonstrating excellent model fit. The scale showed strong internal consistency. Convergent validity was confirmed by positive correlations with VMIQ‐2 and SUIS, while discriminant validity was supported by negligible associations with anxiety and all Heterotrait–Monotrait ratios falling below recommended thresholds. Scalar measurement invariance across gender was established with females scoring slightly higher than males. Finally, age modeling revealed a slight decrease in the vividness of imagery from adolescence to early adulthood, followed by relative stability after that.

**Conclusion:**

The VVIQ‐Pr2 is a psychometrically reliable assessment tool for Persian speakers. In addition to operationalizing subjective visual experience in theories on reality monitoring, it may facilitate future cross‐cultural and developmental research.

## Graphical abstract

We validated the Persian Vividness of Visual Mental Imagery Questionnaire‐2 (VVIQ‐Pr2) in 630 participants, demonstrating its reliability, unidimensional structure, and convergent validity. The VVIQ‐Pr2 provides a robust measure of subjective imagery vividness, supporting cross‐cultural research and theories of perceptual reality monitoring.

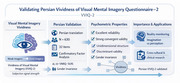

## 1 Introduction

Mental imagery is a basic cognitive mechanism that gives rise to perceptual experiences in the absence of external inputs and forms part of many cognitive processes, such as memory, planning, problem‐solving, emotion regulation, and creativity (Ji et al. 2019; Nanay 1997; Pearson 2019; Pearson et al. 2015). This has important implications for clinical psychology, sport science, education, and cognitive neuroscience (Hicks et al. 2024; Liu et al. 2025; Van Caenegem et al. 2024). One of the salient features of mental imagery is vividness, defined as the clarity and sensory strength of mental images (Pearson 2019). It represents the “signal strength” of internal representations and shows great individual variation, from people who do not experience imagery at all (aphantasia) to individuals with very vivid images (hyperphantasia; Zeman et al. 2020, Zeman et al. 2015). The existence of these poles in the spectra of mental imagery has only recently received scientific attention, in particular through work by Zeman et al. concerning aphantasia and hyperphantasia (Zeman 2024; Zeman et al. 2015).

Prevalence studies put the rate of aphantasia at about 1%, hypophantasia at 3%, and hyperphantasia at 6% of the population (Dance et al. 2022; Wright et al. 2024). Knowing and measuring vividness is crucial for creating a map of human imaginative competence (Dawes et al. 2020). Developmental studies have also reported that across the lifespan, the average level of vividness is not constant, although self‐reported imagery vividness seems to decline from adolescence up to middle age (Gulyás et al. 2022). Generally speaking, older adults report lower vividness of imagery, and it remains an open question how robust these findings are due to issues related to measurement.

The Vividness of Visual Imagery Questionnaire (VVIQ) was developed in 1973 by David Marks, and it is one of the most widely accepted measures of imagery vividness. The original VVIQ consists of 16 items to be used for the assessment of mental imagery, while an extended version, referred to as Persian Vividness of Visual Mental Imagery Questionnaire‐2 (VVIQ‐2), was published in 1995. The latter provided greater item heterogeneity and easier scoring (Marks 1973, 1995). The VVIQ and VVIQ‐2 have been cross‐culturally validated in many studies that showed a high level of internal consistency and good test‐retest reliability (Campos 2011; Campos and Pérez‐Fabello 2009). Factorial analyses performed in many different languages yielded repeatedly a unidimensional factor structure, consistent with the conceptual framework underlying the instrument (Campos et al. 2002; Jankowska and Karwowski 2023). The VVIQ correlates with other imagery scales to provide convergent and discriminant validities. The participants who were known to have vivid imagery, such as artists, tended to score higher in the VVIQ (Jankowska and Karwowski 2023). Consequently, the VVIQ also became the standard measure in research for assessing self‐reported imagery vividness.

Imagery vividness also shares a relation with reality monitoring, which refers to a cognitive process involved in differentiating between actual experiences from imagined ones (Dijkstra, Bosch, and Van Gerven 2019; Dijkstra and Fleming 2023; Johnson, Hashtroudi, and Lindsay 1993) (Dijkstra and Fleming 2023; Johnson et al. 1993). According to traditional theories, the qualitative characteristic of experiences provides a basis for reality monitoring, where real perceptions are stronger and more detailed than imagined ones (Johnson 2006; Johnson et al. 1993; Johnson and Raye 1981). This view suggests that the vividness of experiences can provide evidence regarding whether an experience is real or not. In Perceptual Reality Monitoring theory formalizes this relationship whereby the brain evaluates the strength of internal representations so as to segregate reality from imagination (Dijkstra and Fleming 2023, 2025; Dijkstra et al. 2022). Consistent with this hypothesis, various studies have documented that mental imagery involves the same neural representations as real perception but at a lower level of activity (Pearson 2019; Pearson et al. 2015; Spagna et al. 2021).

However, if mental imagery is vivid, these boundaries tend to blur and can therefore potentially confuse imagined and real experiences. Indeed, research into the populations prone to hallucinations established that intense internal imagery might result in the misattribution of specific experiences, subsequently leading to delusional perception (Allen et al. 2006; Brookwell et al. 2013; Moseley et al. 2016). The high vividness of mental imagery also provoked false memories, thereby hindering the true remembering of events with accuracy (Garry et al. 1996; Gonsalves et al. 2004).

The assessment of imagery vividness is, therefore, important since it explains how we differentiate between fantasy and reality; it also has implications in terms of memory accuracy and mental health (Garrison et al. 2017). The VVIQ may serve as a valuable tool for researching these processes, especially in relation to memory distortions and hallucinations.

Despite the VVIQ's utility, there is currently no validated Persian version of the scale, thus limiting research on mental imagery within Persian‐speaking populations and hindering cross‐cultural research (Campos et al. 2002; Jankowska and Karwowski 2023).). The development of a Persian adaptation involves paying close attention to cultural and linguistic sensitivity in order to ensure appropriate reliability and conceptual equivalence (ITC Guidelines for Translating and Adapting Tests (Second Edition) 2018; Van De Vijver and Tanzer 2004).

Current research seeks to fill this deficit by adapting and validating the VVIQ‐2 for Persian‐speaking populations, testing its psychometric properties. Predictively, it is expected that the Persian VVIQ will reflect a unidimensional structure, high internal consistency, and significant correlations with other imagery scales but weak links to measures of anxiety and personality traits (Campos et al. 2002; Campos and Pérez‐Fabello 2009; Fulford et al. 2018; Roberts et al. 2008; Spiller et al. 2019; Sunday et al. 2017). If this is successfully validated, it could become an important tool in investigating the effects of mental imagery on cognitive function and mental health within Iranian and other Persian‐speaking environments.

## 2 Materials and Methods

### 2.1 Study Design

This research employed a cross‐sectional design to translate, adapt, and validate the Persian version of the Vividness of Visual Imagery Questionnaire‐2 (VVIQ‐Pr2). The study aimed to examine factorial validity, reliability, convergent and divergent validity, and age–gender differences in imagery vividness within a Persian‐speaking population.

### 2.2 Ethics and Reporting

Procedures were in accordance with the Declaration of Helsinki and were approved by Research Ethics Committees of Vice‐Chancellor in Research Affairs—Shahid Beheshti University of Medical Sciences (code: IR.SBMU.RETECH.REC.1404.507; World Medical Association Declaration of Helsinki: Ethical Principles for Medical Research Involving Human Subjects 2013). Participants provided informed digital consent (assent along with parental consent for minors). All subjects were given a prepaid SIM card credit as a gift for participating in the research.

### 2.3 Participants

Participants were recruited through the Porsline online survey platform. The inclusion criteria were being fluent in Persian and provision of informed consent. Exclusion criteria were incomplete responses and patterned responding.

### 2.4 Measures

#### 2.4.1 Sociodemographic Questionnaire

Participants responded to a sociodemographic questionnaire concerning personal characteristics including age, sex, profession, and educational status.

#### 2.4.2 Vividness of Visual Imagery Questionnaire‐2

This VVIQ‐2 is a 32‐item self‐report scale developed to measure the vividness of visual mental imagery. Items fall into eight “scenarios” or “mental scenes,” and each asked participants to imagine certain visual details—things and scenes–and rate the extent to which they are clear or vivid. Items were rated on a 5‐point Likert‐type scale ranging from 1 = No image at all, you only “know” that you are thinking of an object to 5 = Perfectly clear and as vivid as normal vision. Thus, the total score ranges from 32 to 160, with higher scores reflecting more vivid visual imagery. In the original studies, the VVIQ‐2 obtained a high internal consistency (*α* ≈ 0.91) and reached an acceptable convergent validity with other imagery measures (|*r*| ≈ 0.50 with Betts' Questionnaire Upon Mental Imagery or QMI and Object‐Spatial Imagery Questionnaire), while being near‐zero with verbal imagery‐evidence conjoined to support largely unidimensional imagery‐vividness construct (Campos 2011; Campos and Pérez‐Fabello 2009). A translated and culturally adapted Persian version, VVIQ‐Pr2, was used, finalized through standard translation/back‐translation.

#### 2.4.3 Vividness of Movement Imagery Questionnaire‐2

The Vividness of Movement Imagery Questionnaire‐2 (VMIQ‐2) evaluates motor imagery's vividness through three modalities: internal visual imagery (first‐person perspective), external visual imagery (third‐person perspective), and kinesthetic imagery (sensation of movement). The 36‐item scale is rated on a 5‐point scale, with scores reflecting clarity of imagery. The Persian version has been validated among Iranian athletes, showing Cronbach's alpha coefficients of ∼0.86, 0.89, 0.91, and 0.95 for various subscales. Concurrent validity with another questionnaire and factor analyses confirmed the structure (Rostami Haji Abadi et al. 2011). As would be expected, internal consistencies were similarly high in the present study, with α values of [0.94, 0.94, 0.95, and 0.97] for the three subscales and total scale and *ω* values of [0.96, 0.97, 0.97, and 0.98].

#### 2.4.4 Spontaneous Use of Imagery Scale

The Spontaneous Use of Imagery Scale (SUIS) is a 12‐item self‐report measure assessing the frequency of spontaneous mental imagery in daily life, rated on a 5‐point scale (Reisberg et al. 2003). The Persian version has good internal consistency, Cronbach's alpha = 0.75 (Naderi Rajeh et al. 2023). In our sample, reliability was comparable, with *α* = [0.82] and *ω* = [0.88].

#### 2.4.5 Generalized Anxiety Disorder Scale

The Generalized Anxiety Disorder Scale (GAD‐7) is a scale for generalized anxiety symptoms over a 2‐week period assessed with seven items rated on a 0–3 scale. The overall score ranges from 0 to 21, reflecting the severity of symptoms (Spitzer et al. 2006). The Persian version has been validated among Iranian populations and has shown excellent internal consistency, *α* = 0.85, and good convergent validity (Naeinian et al. 2011). The results from our study show strong internal consistency, confirming *α* = 0.88 and *ω* = 0.93, which conforms to previous findings on its reliability and validity.

#### 2.4.6 Ten‐Item Personality Inventory

The Ten‐Item Personality Inventory (TIPI) is a brief self‐report measure of the Big Five personality traits: extraversion, agreeableness, conscientiousness, emotional stability, and openness to new experiences. It is composed of 10 items, two for each trait, rated on a 7‐point Likert scale. While it prioritizes brevity over depth, the scale has been validated against the standard Five Factor Inventory (NEO) (Mohammad Zadeh and Najafi 2010; Rammstedt and John 2007). In our sample, internal consistency—expectedly modest for two‐item subscales—was from *α* = 0.25 to *α* = 0.55 (polychoric *α* from 0.33 to 0.60). Overall, the TIPI behaved as a very brief index of broad trait content, suitable for covariate and divergent‐validity purposes, with the expected trade‐off in internal consistency.

### 2.5 Translation, Adaptation, and Data Analysis

Translation was carried out based on established standards for cross‐cultural adaptation (Beaton et al. 2000). These two translations were then combined into one consensus Persian translation by a committee. After this step, the agreed translation was back‐translated into English by a bilingual translator who had never seen the original questionnaire. Finally, the research team compared the back‐translation with the original VVIQ‐2 for semantic, idiomatic, and conceptual equivalence and made necessary adjustments. The Persian version closely resembles the original VVIQ‐2 in terms of factor structure and item content, with no changes made to the conceptual meaning of any items. All modifications were solely linguistic and cultural in nature, with the goal of maintaining semantic equivalency and comprehensibility for respondents who speak Persian. The Supporting Information contains the Persian scale that was employed in this investigation.

All the statistical analyses were carried out in R using the following packages: lavaan for factor analysis, psych to calculate reliability, and semTools for other analyses (Jorgensen et al. 2012; Revelle 2007; Rosseel et al. 2012). Comprehensive details about the analyses and code can be found on the GitHub repository (https://github.com/atashrooz/VVIQ_Validity.git).

Three confirmatory factor analyses were conducted using the weighted least squares mean and variance (WLSMV) estimator to test the factorial structure of a scale with 32 items related to visual imagery vividness (Brauer et al. 2023). The first analysis implemented a unidimensional model where all items loaded onto one latent factor. The second model reflected the VVIQ's design, grouping items into eight scenes, with item residuals within each scene assumed to correlate due to shared contextual priming while still having a general factor (Marks 1995). A bifactor model was also evaluated, positing a general factor alongside scene‐specific group factors, addressing the hierarchical questionnaire structure and the potential for scene‐specific variance to reveal meaningful subdimensions of imagery vividness (Reise 2012). Three models were compared to each other to identify which model best represented the data's structure. The following model fit indices were used to investigate model fit: *χ* ^2^, comparative fit index (CFI), Tucker–Lewis index (TLI), root mean square error of approximation (RMSEA), and standardized root mean square residual (SRMR), where good fit was defined as CFI/TLI ≥ 0.95, RMSEA ≤ 0.05, and SRMR ≤ 0.08 (Hu and Bentler 1999; Schermelleh‐Engel et al. 2003).

A multiple‐group confirmatory factor analysis based on ordinal item responses modeled using the WLSMV adjusted estimator was performed to investigate the factorial structure of the VVIQ across gender groups. The analysis involved robust *χ* ^2^ values and *χ* ^2^ difference testing for nested models. A sequence of one‐factor models was tested for males and females, starting with configural invariance allowing the free estimation of item thresholds and factor loadings. Next, metric invariance was achieved by constraining item thresholds across groups, followed by scalar invariance through additional constraints on factor loadings. Several indices, including *χ* ^2^, CFI, TLI, RMSEA, and SRMR, were used to assess the model's fit (Chen 2007). Once scalar invariance was established, latent mean gender differences were estimated by estimating the mean of the male group against zero and fixing the latent mean of the female group to zero.

The reliability of the tools was assessed with Cronbach's *α* and McDonald's ω. These measures were calculated for the scales to estimate internal consistency.

The evaluation of convergent and discriminant validity involved analyzing the correlation patterns between the total VVIQ‐Pr2 score and scores from other validated measures. A positive correlation was anticipated between the VVIQ, VMIQ, and SUIS, with Pearson's *r* or Spearman's *ρ* used for correlation calculations. Discriminant validity was assessed through correlations with distinct constructs and the Heterotrait–Monotrait ratio (HTMT), hypothesizing an HTMT value below 0.85 for the GAD‐7 and TIPI (Henseler et al. 2015). Gender variation in VVIQ‐Pr2 scores was explored using an independent samples *t*‐test, checking for the homogeneity of variances with Levene's test and reporting Welch's *t*‐test as necessary. Effect sizes for *t*‐tests were calculated using Cohen's *d* (Cohen 2013).

Finally, the relationship between imagery vividness and age was investigated through regression models. Nonlinear associations were evaluated using generalized additive models (GAMs), with model comparisons conducted against linear and quadratic specifications.

## 3 Results

### 3.1 Descriptive Statistics

**FIGURE 1:**
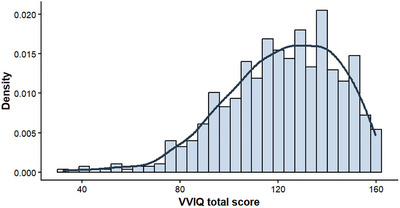
The response distribution for the translated VVIQ‐2.

There were 630 participants in the final analytic sample (74.9% female; mean age = 28.4 years, SD = 8.7, max = 68, min = 14). The majority had a bachelor's degree or higher (53.3%) and were single (85%). The Supporting Information contains descriptive statistics for each of the research variables (Tables S1–S8; Figure 1).

### 3.2 Confirmatory Factor Analysis

As noted in Section 2, three confirmatory factor analysis (CFA) models were estimated. First, a unidimensional model was specified in which all 32 items loaded onto a single latent factor for general visual imagery vividness. The data were not well fitted by this initial model (see Table 1). Significant misfit was indicated by the elevated RMSEA and SRMR values, despite the fact that the comparative fit indices (CFI, TLI) were adequate. This demonstrated that the unidimensional model was insufficient.

**TABLE 1:** CFA model fit measures comparison for VVIQ‐Pr2.

Model	*χ* ^2^	*df*	CFI	TLI	RMSEA [95% CI]	SRMR	ΔCFI	ΔRMSEA
Unidimensional + correlated residuals	920.95	416	0.997	0.996	0.031 [0.027, 0.035]	0.041	—	—
Unidimensional	3769.17	464	0.961	0.96	0.105 [0.101, 0.108]	0.080	0.036	‐ 0.074
Bifactor	1057.12	440	0.996	0.995	0.035 [0.031, 0.039]	0.043	0.001	‐ 0.004

A second model was specified to represent the test's design in which the responses from items within a scene were assumed to have common residual variance above and beyond the general factor because they share contextual priming. The second model therefore had the same one general factor but also allowed the item residuals within each of the eight scenes to correlate. A significant better and superior fit to the data was obtained with this model specification (see Table 1). Every item had a high and statistically significant factor loading to the general factor (see Table S9).

Finally, a bifactor model was tested to examine whether an additional layer of domain‐specific (scene‐based) factors could explain item covariance beyond the general vividness factor. In this model, all items loaded on a general imagery vividness factor as well as on one of eight orthogonal scene‐specific group factors. Although the bifactor model demonstrated good fit indices, its performance was not meaningfully superior to the correlated residual model (ΔCFI = 0.001; ΔRMSEA = −0.004). This suggests that introducing explicit scene factors did not substantially improve model fit beyond what was achieved by modeling correlated residuals. Given the principle of parsimony, the unidimensional model with correlated residuals was therefore preferred, as it provided an equally strong yet more conceptually and statistically economical representation of the data. This suggests that a single latent vividness factor adequately captures the structure of visual imagery experience once local dependencies among items within scenes are accounted for.

In summary, the CFA preferred a unidimensional model of visual imagery vividness, but only when local dependencies among items that share the same mental scene were held constant. Such a model with correlated residuals across scenes fit the data very well.

### 3.3 Reliability

Internal scale consistency was excellent. Cronbach's α was 0.94 for the 32‐item scale. McDonald's ω, using polychoric correlations, was 0.96. These results confirm the Persian VVIQ's high internal consistency.

### 3.4 Convergent and Discriminant Validity

Prior to validity testing, assumptions for correlation analyses were examined. Shapiro–Wilk tests indicated that all three variables deviated from normality (VVIQ, *W* = 0.96, *p* < 0.001; VMIQ, *W* = 0.96, *p* < 0.001; SUIS, W = 0.991, *p* < 0.001). Thus, nonparametric Spearman's rank‐order correlations were employed for all analyses for strength.

**FIGURE 2:**
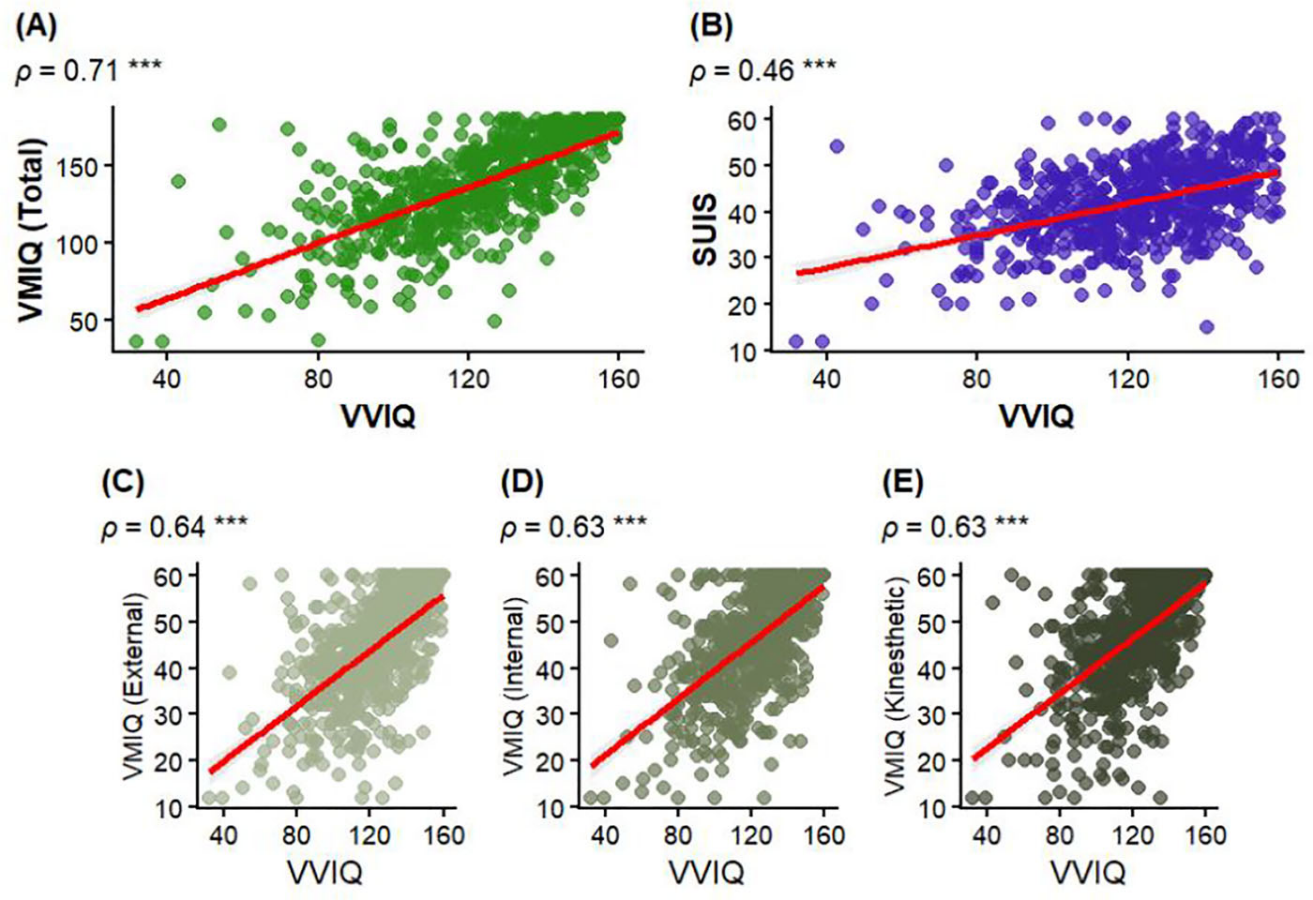
Convergent validity of the VVIQ‐2Pr. Scatterplots show associations between VVIQ total score and (A) VMIQ total score, (B) SUIS, and VMIQ subscales: (C) External, (D) Internal, and (E) Kinesthetic imagery. Each point represents one participant; the red line is the least‐squares fit with 95% CI shading. Spearman rank correlations (*ρ*) and significance are printed in each panel (***p < 0.001). Axes display raw summed scores.

The VVIQ‐2Pr exhibited a strong positive correlation with the VMIQ. The VVIQ‐2Pr exhibited a positive correlation with the SUIS. VMIQ subscales showed strong positive correlations with External Visual Imagery, Internal Visual Imagery, and Kinesthetic Imagery as well (Figure 2). All these associations retained significance after a Bonferroni correction for multiple comparisons (adjusted *p* < 0.001). The robust parallel construct correlations provide strong evidence of convergent validity for the VVIQ‐2Pr. The significant positive correlation between the VVIQ and VMIQ suggests strong convergent validity because both instruments measure similar constructs related to mental imagery vividness. The moderate positive correlation between the VVIQ and SUIS is indicative of convergent validity because both measures share substantial variance in the assessment of mental imagery.

Discriminant validity was tested with correlations of the VVIQ and theoretically unrelated concepts measures: anxiety (GAD‐7) and the Big Five personality factors of the TIPI. Assuming before analysis, the assumptions were tested. Shapiro–Wilk tests showed that all variables were not normally distributed (all *p* < 0.001), and therefore Spearman's rank‐order correlation was used. Interquartile range (IQR) approach to analyzing outliers did not show any considerable number of possible outliers (0%–4.4% per item), which did not have any significant impact on the result. Homoscedasticity was confirmed for the core relationship of the VVIQ and GAD‐7 using a nonsignificant Breusch–Pagan test (BP = 1.44, *p* = 0.231). The correlations between the VVIQ and the divergent validity measures are presented in Table 2. Bonferroni correction was applied to account for multiple comparisons.

**TABLE 2:** Correlations between the VVIQ‐Pr2, GAD‐7 and TIPI.

Measure	Spearman's *ρ*	*p* value	Bonferroni‐adjusted *p*
GAD‐7	−0.07	0.086	0.51
Extraversion	0.13	<0.001	0.004
Agreeableness	−0.007	0.86	1
Conscientiousness	0.26	<0.001	< 0.001
Emotional stability	0.18	<0.001	< 0.001
Openness	0.28	<0.001	< 0.001

Among the six correlations tested, four were statistically significant. As expected, the VVIQ correlated trivially and non‐significantly with the GAD‐7, providing robust evidence for divergent validity with anxiety. When correlated with agreeableness as well, it was negligible and nonsignificant, demonstrating divergent validity once more. Conversely, the remaining personality traits—extraversion, conscientiousness, emotional stability, and openness—showed positive correlations. Extraversion and emotional stability were weak, while conscientiousness and openness were moderate. This pattern of results provides acceptable support for divergent validity for the VVIQ. Associations with Extraversion, Conscientiousness, Emotional Stability, and particularly Openness were theoretically coherent, suggesting partial overlap without compromising discriminant validity.

To further determine discriminant validity, the Heterotrait–Monotrait ratio of correlations was determined. All HTMT ratios between VVIQ and the other constructs were below the conservative cut‐off of 0.85; they varied between 0.05 and 0.52. This indicates sufficient discriminant validity in each instance. That the higher values were with Agreeableness at 0.47, Conscientiousness at 0.52, and Openness at 0.49 means larger similarity with those traits. The trend suggests that while statistically a discriminant validity is possible, the Conscientiousness and Openness constructs are more conceptually similar to visual imagery vividness compared to the remaining tests, consistent with the findings of the correlation (see Table 3).

**TABLE 3:** Discriminant validity via HTMT ratios.

	VVIQ	GAD	EX	AG	CO	EM	OP
VVIQ	1						
GAD	0.05	1					
EX	0.12	0.07	1				
AG	0.43	0.10	0.47	1			
CO	0.52	0.27	0.55	0.73	1		
EM	0.25	0.69	0.17	0.51	0.45	1	
OP	0.43	0.13	0.33	0.75	0.75	0.36	1

### 3.5 Measurement Invariance and Group Modelling

A unidimensional VVIQ model demonstrated an acceptable configural fit for both females and males, and setting thresholds equal did not worsen the model fit. Equal loadings were added. Changes met Chen's (2007) criteria (|ΔCFI| ≤ 0.001; |ΔRMSEA| ≤ 0.006; ΔSRMR ≈ 0.000), thereby supporting threshold and loading invariance and allowing latent‐mean comparisons by gender.

**FIGURE 3:**
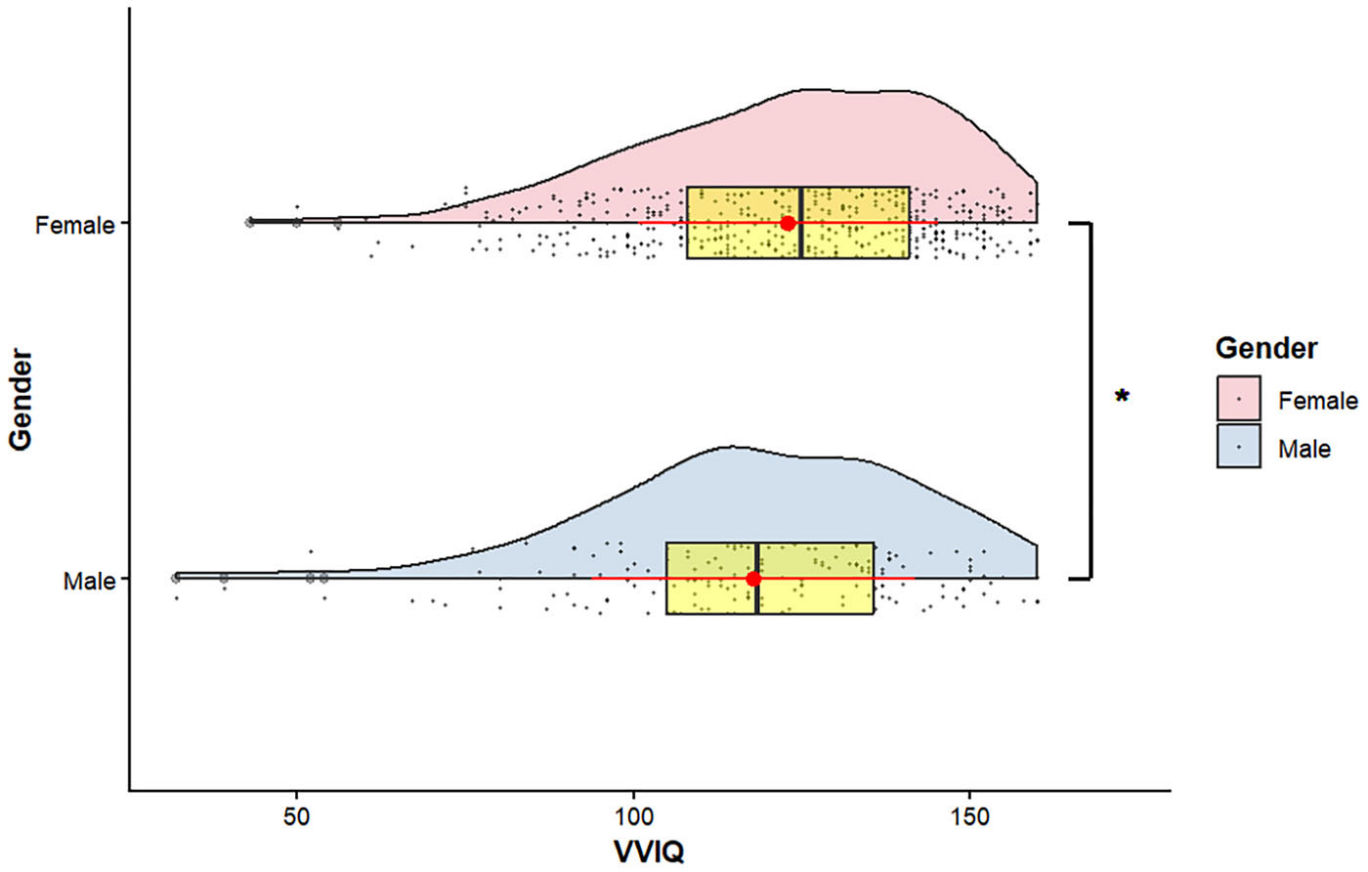
VVIQ by gender—distribution and group difference. Half‐violin plots with individual observations (gray dots), overlaid boxplots (yellow; median and IQR), and mean ± SD (red point with whiskers). The bracket denotes the Welch two‐sample *t*‐test; * sign defines *p* < 0.05 (exact value reported in text). Axis x shows VVIQ scores.

We examined whether the VVIQ measurement was invariant across gender. As shown in Table 4, the decline in fit when comparing more restricted models with less restricted ones (metric vs. configural; scalar vs. metric) was negligible relative to standard criteria. Having established measurement invariance, the VVIQ allows for unbiased comparison of scores across genders. In line with prior research, we found that women scored slightly but significantly higher than men (latent mean difference = −2.38, 95% CI [−9.47, −0.90], *p* = 0.02; Figure 3).

**TABLE 4:** Measurement invariance across gender.

Model	*χ* ^2^	df	CFI	TLI	RMSEA [95% CI]	SRMR	ΔCFI	ΔRMSEA
Configural	1347.37	832	0.937	0.925	0.049 [0.044, 0.053]	0.045	—	—
Metric (thresholds)	1371.67	863	0.938	0.929	0.047 [0.043, 0.052]	0.051	+0.001	−0.002
Scalar (thresholds + loadings)	14110.07	894	0.937	0.930	0.047 [0.042, 0.052]	0.052	−0.001	0.000

**FIGURE 4:**
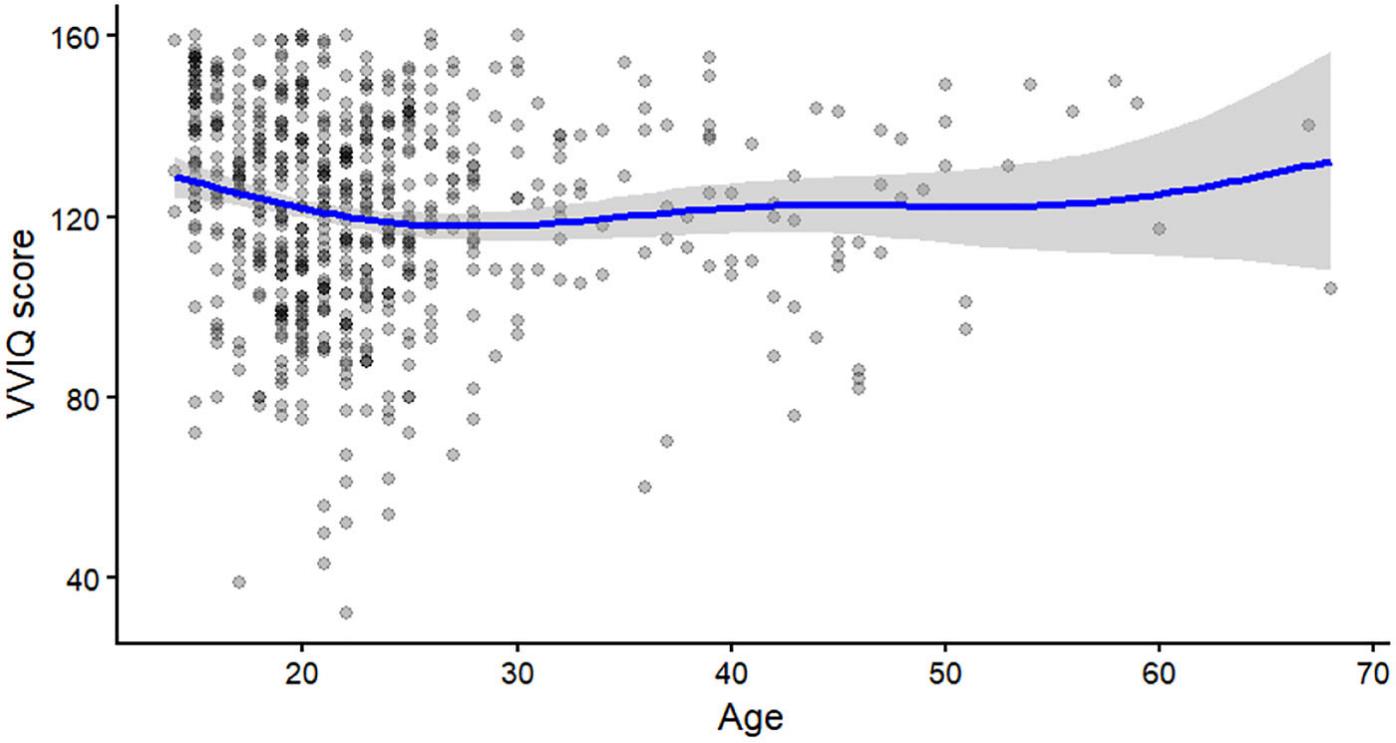
Age‐related variation in vividness of visual mental imagery (VVIQ score). Each point represents an individual participant. The solid line depicts the fitted penalized generalized additive model (GAM) smooth, with the shaded area indicating the 95% confidence interval.

To test whether the vividness of mental imagery varied with age, we analyzed age as a continuous predictor of VVIQ scores. Model comparisons showed that a GAM with AIC = 5699.14 outperformed both linear (AIC = 5732.34) and quadratic (AIC = 5724.82) models. In this GAM, the smooth age term was statistically significant, *F* = 5.79, *p* < 0.001, although the proportion of variance explained was small, adjusted *R* ^2^ = 0.059. Such findings indicate a modest but reliable nonlinear association between age and imagery vividness, with a slight decline in vividness during adolescence into early adulthood and remaining relatively stable thereafter (Figure 4).

Overall, the vividness of imagery tends to decrease moderately from adolescence to young adulthood but remains stable across the various ages, as evident from the analyses involving the continuous variable of age. Additionally, the exploratory analyses based on age categories did not reveal statistically significant differences among the older participants. These results are to be treated with caution owing to the smaller sample size of older participants, thus necessitating increased samples of older participants in future studies.

## 4 Discussion

The validity and reliability of the VVIQ‐2 were examined in a Persian‐speaking population after it was translated into Persian, demonstrating the tool's significance for research. Key findings have been emphasized: (1) imagery vividness is best characterized by a unidimensional structure; (2) a distinctive discriminant validity linking vivid imagery to personality traits, especially Openness to Experience; and (3) gender and lifespan variations in imagery vividness within a new cultural context.

### 4.1 Summary of Psychometric Properties

The psychometric evaluation showed strong internal consistency: *α* = 0.94, *ω* = 0.96. Correlational analyses revealed convergent validity with the VMIQ‐2 and SUIS, showing significant differences in imagery quality versus frequency by the VVIQ and SUIS, respectively (Dahm 2022; Marks and Isaac 1995; Yamaki 2025). A unidimensional substantive factor with method variance was confirmed by a one‐factor model that included scene‐specific residual correlations and showed good fit while retaining strong primary loadings (Campos 2011; Campos et al. 2002; Jankowska and Karwowski 2023). Discriminant validity was adequate; the results showed that vivid imagery is linked to openness to experience while still being distinct from more general personality traits. The specificity of the VVIQ‐2 was not compromised by the small but significant correlations that were found with some of the Big Five dimensions. These correlations were theoretically consistent. Overall, the VVIQ‐Pr2 shows validity and reliability as a useful instrument for evaluating the vividness of imagery.

### 4.2 Convergent and Discriminant Validity: Imagery Vividness and the Open Personality

As expected, the VVIQ‐Pr2 showed convergent validity, as indicated by its high correlation with the VMIQ‐2 and its moderate correlation with the SUIS; Campos and Pérez‐Fabello 2009; Commodari et al. 2024; Roberts et al. 2008). According to this pattern, people with strong visual imagery are also likely to have strong kinesthetic and motor imagery, and they are also more likely to use imagery on occasion, in the moment when engaging in daily activities.

However, a theoretically more nuanced picture emerged from the discriminant validity findings. Associations with Big Five traits were small to moderate—highest for Openness and Conscientiousness, followed by Extraversion and Emotional Stability—whereas relations with Agreeableness and the GAD‐7 were essentially null. The HTMT with Openness remained below our discriminant‐validity threshold, and HTMT values for the other traits were also below threshold. Together with the strong convergence with the VMIQ‐2 and SUIS, this pattern indicates that the VVIQ‐Pr2 captures imagery vividness without collapsing into broad personality domains.

This trend challenges a reductionist account of imagery vividness as a standalone domain‐general cognitive module. Instead, it suggests that vivid mental imagery skill can be supported by a broad cognitive‐affective personality factor with the attributes of curiosity, imagination, and appreciation for inner experience (Jankowska and Karwowski 2023). The relation with Conscientiousness could be the deployment of elaborate imagery in goal‐striving action and planning. These findings promote the reconceptualization of imagery vividness alone, but as a dimension that enters into interactions with central personality dimensions to shape one's internal world and degree of involvement in it.

### 4.3 Lifespan and Gender Patterns in Imagery Vividness

Our study also uncovered significant demographic variation. We ensured strict measurement invariance across gender, which allowed for the performance of fair comparisons of means. Invariance across gender was supported, permitting mean comparisons. Women reported slightly but significantly more vivid imagery than men, which is in line with an increasing amount of international research (Cui et al. 2007; Jankowska and Karwowski 2023; Richardson 1995). This small but consistent difference on the gender axis may reflect the neurodevelopmental or socio‐cognitive processes underlying how men and women subjectively evaluate their internal representations.

According to our findings, the vividness of imagery, decreased slightly in young adulthood, and then remained constant. A GAM model outperformed linear and quadratic models, with a modest peak in adolescence. Despite its slight magnitude, this curvilinear pattern is in line with developmental models that indicate the peak sensory and imaginative capacities occur in the early stages of adolescence, possibly followed by a decline in processes toward an abstract or gist‐based mode in adulthood (Gulyás et al. 2022; Parker and Lovell 2012; Subirats et al. 2018). Given the small sample size, it is important to interpret the lack of a sharp decline in our older adult sample with caution, but it indicates that the fundamental capacity for productive imagery is largely maintained throughout mid‐ to late‐life.

### 4.4 Cross‐Cultural and Theoretical Implications: Reality Monitoring

The validation of the VVIQ‐Pr2 opens new opportunities for cross‐cultural mental imagery research by enabling direct comparison with the sizable global sample of Persian‐speaking samples. In the potential clinical utilities, the tool can be used to investigate the role of mental imagery in psychopathologies, such as trauma‐related disorders where intrusive imagery is a key symptom. Curious questions concerning the function of imagery in creativity and aesthetic appreciation within this cultural group are also brought up by the association between vividness and openness.

Our results provide tools for exploring frameworks positing that imagery vividness reflects a form of “subjective signal strength”: richer, more detailed internal representations feel more percept‐like (Dijkstra and Fleming 2023). This has implications for reality monitoring—the process of distinguishing internally generated from externally sourced events (Dijkstra et al. 2025).

### 4.5 Limitations and Future Directions

There are several limitations of this research that point to clear directions for the future. Our sample was young, highly educated, and not older or rural in diversity. Future research can use more representative and clinical samples to derive norms and assess clinical application. Further, employing only self‐report measures is open to bias. Cross‐validation of our findings with behavioral tasks, neural imaging, and eye‐tracking would provide a firmer basis. Finally, the link between the personality dimension of Openness and rich imagery suggests that certain cognition processes, including fantasy absorption, can be critical, so we propose that these processes be investigated further.

## 5 Conclusion

The VVIQ‐Pr2 is a valid and reliable tool that will enable the systematic study of mental imagery in Persian speakers, to sum up. The findings of this study go beyond simple translation and offer new information about the vividness of imagery's structure and its intriguing relationship to personality and development. We have shown that the “mind's eye” is not a culturally specific phenomenon, but rather a universal cognitive ability.

## Author Contributions

**Mohammad Atashrooz**: conceptualization, writing – original draft, formal analysis, visualization, methodology, validation, resources. **Fatemeh Mirzai**: data curation, project administration, conceptualization, methodology, writing – review and editing. **Maede Amin Roaya**: data curation, methodology, writing – review and editing. **Hannaneh Fayyaz Rouhi**: data curation, writing – review and editing. **Arash Ghadir**: data curation, Writing – review and editing. **Hoda Doosalivand**: supervision, writing – review and editing. **Amir Sam Kianimoghadam**: supervision, writing – review and editing, investigation.

## Funding

This study is funded by the Student Research Committee, Shahid Beheshti University of Medical Sciences, Tehran, Iran. (Grant NO. 1404/36650)

## Conflicts of Interest

The authors declare no conflict of interest.

## Supporting information




**Supplementary Material**: brb371203‐sup‐0001‐SuppMat.docx

## Data Availability

The datasets analyzed for this study can be found in the GitHub repository named VVIQ_Validity (https://github.com/atashrooz/VVIQ_Validity.git).
