# Supplementary material for: Measuring the Subjective Signal Strength: Validating Persian Vividness of Visual Mental Imagery Questionnaire‐2
Source: Brain Behav. 2026 Jan 15;16(1):e71203. doi: 10.1002/brb3.71203 (PMC12808923; doi:10.1002/brb3.71203)
Supplement: Supplementary file 1 — Supplementary Material: brb371203‐sup‐0001‐SuppMat.docx [file BRB3-16-e71203-s001.docx]

Supplementary Material

# Supplementary Figures and Tables

## Supplementary Figures:


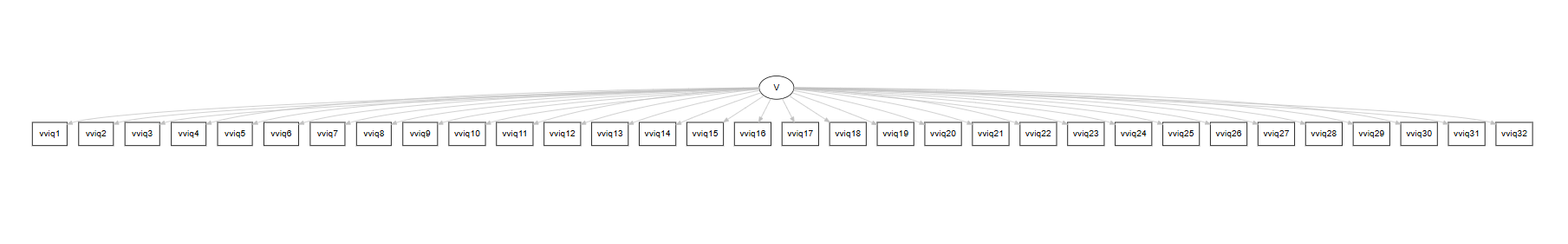


Supplementary Figure 1. Unidimensional Model.


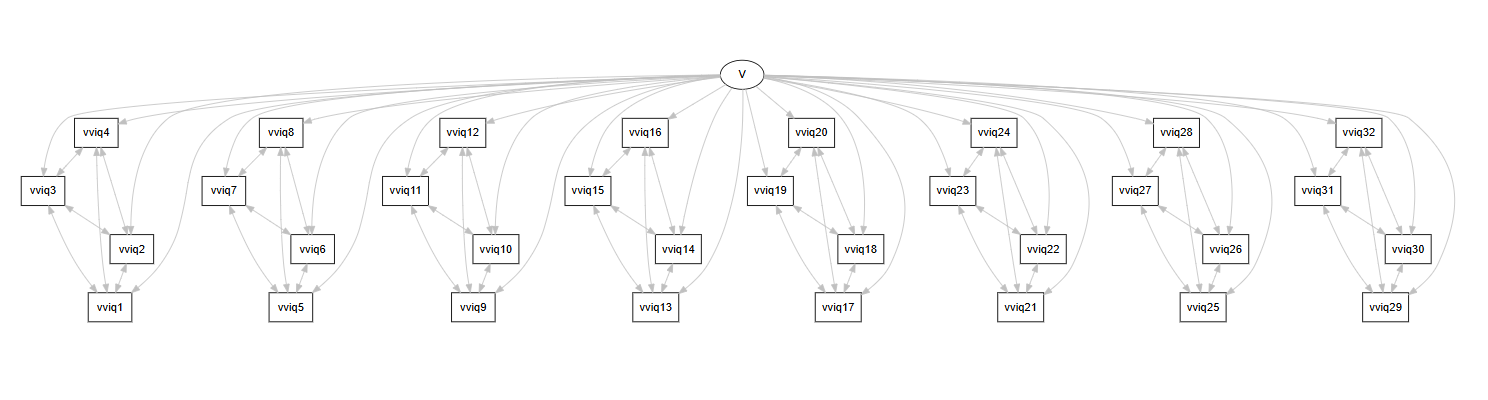


Supplementary Figure 2. Unidimensional + Correlated Residuals Model (Final Model)


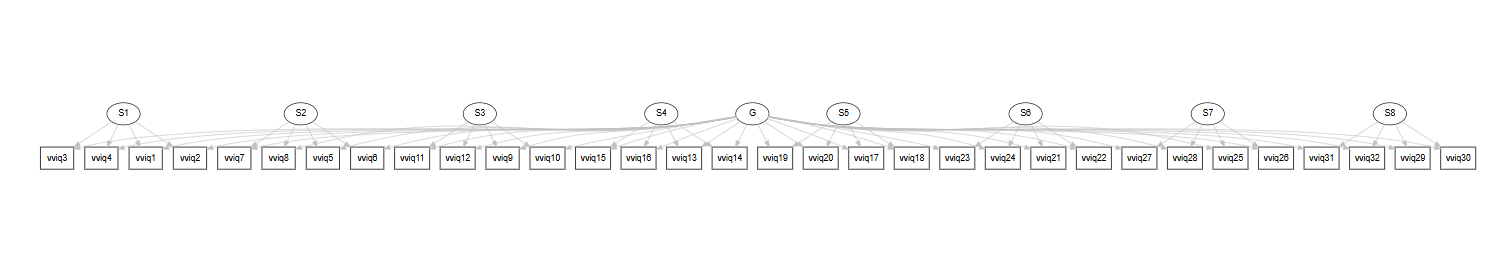


Supplementary Figure 3. Bifactor Model

## Supplementary Tables

Supplementary Table 1. Descriptive statistics of the sample – Gender.

| **Gender** | | **Frequency** | | **Percentage** | |  |
| --- | --- | --- | --- | --- | --- | --- |
| Female |  | 472 |  | 74.9 |  |  |
| Male |  | 158 |  | 25.1 |  |  |
| Total |  | 630 |  | 100 |  |  |

Supplementary Table 2. Descriptive statistics of the sample – Marital Status.

| **Gender** | | **Frequency** | | **Percentage** | |  |
| --- | --- | --- | --- | --- | --- | --- |
| Single |  | 540 |  | 85.7 |  |  |
| Married |  | 90 |  | 14.3 |  |  |
| Total |  | 630 |  | 100 |  |  |

Supplementary Table 3. Descriptive statistics of the sample – Age groups.

| **Age Groups** | **N** | **Mean** | **SD** | **Median** |
| --- | --- | --- | --- | --- |
| Adolescents | 145 | 129 | 21.4 | 132 |
| Young Adults | 269 | 118 | 23.6 | 120 |
| Adults | 80 | 125 | 18.5 | 125 |
| Older Adults | 31 | 119 | 21.4 | 124 |

Supplementary Table 4. Descriptive statistics of the sample – Educational Degree.

| **Education** | | **Frequency** | | **Percentage** | |  |
| --- | --- | --- | --- | --- | --- | --- |
| Not Educated |  | 3 |  | 0.5 |  |  |
| Primary School |  | 0 |  | 0.0 |  |  |
| Middle School |  | 68 |  | 10.8 |  |  |
| High School |  | 180 |  | 28.6 |  |  |
| Associate Degree |  | 43 |  | 6.8 |  |  |
| Bachelor Degree |  | 218 |  | 34.6 |  |  |
| Master Degree |  | 103 |  | 16.3 |  |  |
| PhD Degree |  | 15 |  | 2.4 |  |  |
| Post Doc |  | 0 |  | 0.0 |  |  |
| Total |  | 630 |  | 100.00 |  |  |

Supplementary Table 5. Descriptive statistics of the sample – Civil Status.

| **Civil status** | | **Frequency** | | **Percentage** | |  |
| --- | --- | --- | --- | --- | --- | --- |
| Urban |  | 558 |  | 89.9 |  |  |
| Rural |  | 53 |  | 8.5 |  |  |
| Suburbanite |  | 10 |  | 1.6 |  |  |
| Total |  | 630 |  | 100 |  |  |

Supplementary Table 6. Descriptive statistics of the sample – Employed/study situation.

| **Job situation** | | **Frequency** | | **Percentage** | |  |
| --- | --- | --- | --- | --- | --- | --- |
| Student |  | 403 |  | 64.0 |  |  |
| Employed |  | 200 |  | 31.7 |  |  |
| Unemployed |  | 27 |  | 4.3 |  |  |
| Total |  | 630 |  | 100.00 |  |  |

Supplementary Table 7. Descriptive statistics of the main variables.

|  | **Mean** | **SD** | **IQR** | **Skewness** | **SE** | **Kurtosis** | **SE** | **25th percentile** | **50th percentile** | **75th percentile** |
| --- | --- | --- | --- | --- | --- | --- | --- | --- | --- | --- |
| VVIQ | 121.68 | 22.82 | 33 | -0.63 | 0.1 | 0.3 | 0.2 | 107 | 124 | 140 |
| VMIQ | 137.32 | 29.77 | 42.75 | -0.62 | 0.1 | 0.09 | 0.2 | 118.25 | 139 | 161 |
| SUIS | 42.07 | 8.27 | 11 | -0.26 | 0.1 | 0.13 | 0.2 | 37 | 42 | 48 |
| GAD-7 | 10.14 | 5.42 | 8 | 0.1 | 0.1 | -0.9 | 0.2 | 6 | 10 | 14 |
| TIPI Extraversion | 3.91 | 1.68 | 2.5 | 0.06 | 0.1 | -0.82 | 0.2 | 2.5 | 4 | 5 |
| TIPI Agreeableness | 4.58 | 1.05 | 1 | 0.25 | 0.1 | 0.02 | 0.2 | 4 | 4.5 | 5 |
| TIPI Conscientiousness | 5.23 | 1.26 | 1.88 | -0.33 | 0.1 | -0.56 | 0.2 | 4.5 | 5.5 | 6.38 |
| TIPI Emotional Stability | 3.85 | 1.46 | 2 | 0.04 | 0.1 | -0.48 | 0.2 | 3 | 4 | 5 |
| TIPI Openness | 5.28 | 1.32 | 2 | -0.6 | 0.1 | -0.12 | 0.2 | 4.5 | 5.5 | 6.5 |

Note. VVIQ = Vividness of Visual Imagery Questionnaire, VMIQ = Vividness of Motor Imagery Questionnaire, SUIS = Spontaneous Use of Imagery Scale, GAD = Generalized Anxiety Disorder, TIPT = Ten Item Personality Inventory.

| **Supplementary Table 8.** Descriptive statistics of the items in the VVIQ_2Pr | | | | | | | | | | | | | | | | | | | | | | | |
| --- | --- | --- | --- | --- | --- | --- | --- | --- | --- | --- | --- | --- | --- | --- | --- | --- | --- | --- | --- | --- | --- | --- | --- |
|  | | **Mean** | | **SD** | | **Skewness** | | **SE** | | **Kurtosis** | | **SE** | | **Shapiro-Wilk** | | ***p*-value of Shapiro-Wilk** | | **25th percentile** | | **50th percentile** | | **75th percentile** | |
| Vviq 1 |  | 4.08 |  | 1.16 |  | -1.25 |  | 0.1 |  | 0.69 |  | 0.2 |  | 0.76 |  | < .001 |  | 4 |  | 4 |  | 5 |  |
| Vviq 2 |  | 3.98 |  | 1.09 |  | -1.02 |  | 0.1 |  | 0.42 |  | 0.2 |  | 0.82 |  | < .001 |  | 3 |  | 4 |  | 5 |  |
| Vviq 3 |  | 3.72 |  | 1.24 |  | -0.75 |  | 0.1 |  | -0.42 |  | 0.2 |  | 0.85 |  | < .001 |  | 3 |  | 4 |  | 5 |  |
| Vviq 4 |  | 3.94 |  | 1.19 |  | -0.96 |  | 0.1 |  | -0.05 |  | 0.2 |  | 0.81 |  | < .001 |  | 3 |  | 4 |  | 5 |  |
| Vviq 5 |  | 3.55 |  | 1.17 |  | -0.53 |  | 0.1 |  | -0.55 |  | 0.2 |  | 0.89 |  | < .001 |  | 3 |  | 4 |  | 4 |  |
| Vviq 6 |  | 3.91 |  | 1.17 |  | -0.93 |  | 0.1 |  | 0.02 |  | 0.2 |  | 0.82 |  | < .001 |  | 3 |  | 4 |  | 5 |  |
| Vviq 7 |  | 3.79 |  | 1.26 |  | -0.77 |  | 0.1 |  | -0.5 |  | 0.2 |  | 0.84 |  | < .001 |  | 3 |  | 4 |  | 5 |  |
| Vviq 8 |  | 3.87 |  | 1.2 |  | -0.86 |  | 0.1 |  | -0.22 |  | 0.2 |  | 0.83 |  | < .001 |  | 3 |  | 4 |  | 5 |  |
| Vviq 9 |  | 4 |  | 1.17 |  | -1.02 |  | 0.1 |  | 0.14 |  | 0.2 |  | 0.8 |  | < .001 |  | 3 |  | 4 |  | 5 |  |
| Vviq 10 |  | 3.84 |  | 1.08 |  | -0.72 |  | 0.1 |  | -0.15 |  | 0.2 |  | 0.86 |  | < .001 |  | 3 |  | 4 |  | 5 |  |
| Vviq 11 |  | 3.92 |  | 1.07 |  | -0.82 |  | 0.1 |  | 0.01 |  | 0.2 |  | 0.84 |  | < .001 |  | 3 |  | 4 |  | 5 |  |
| Vviq 12 |  | 4.14 |  | 1.11 |  | -1.24 |  | 0.1 |  | 0.7 |  | 0.2 |  | 0.76 |  | < .001 |  | 4 |  | 5 |  | 5 |  |
| Vviq 13 |  | 3.8 |  | 1.11 |  | -0.64 |  | 0.1 |  | -0.4 |  | 0.2 |  | 0.86 |  | < .001 |  | 3 |  | 4 |  | 5 |  |
| Vviq 14 |  | 3.98 |  | 1 |  | -0.83 |  | 0.1 |  | 0.19 |  | 0.2 |  | 0.84 |  | < .001 |  | 3 |  | 4 |  | 5 |  |
| Vviq 15 |  | 3.91 |  | 1.09 |  | -0.9 |  | 0.1 |  | 0.18 |  | 0.2 |  | 0.84 |  | < .001 |  | 3 |  | 4 |  | 5 |  |
| Vviq 16 |  | 3.75 |  | 1.2 |  | -0.72 |  | 0.1 |  | -0.39 |  | 0.2 |  | 0.86 |  | < .001 |  | 3 |  | 4 |  | 5 |  |
| Vviq 17 |  | 3.5 |  | 1.23 |  | -0.35 |  | 0.1 |  | -0.9 |  | 0.2 |  | 0.89 |  | < .001 |  | 3 |  | 4 |  | 5 |  |
| Vviq 18 |  | 3.34 |  | 1.29 |  | -0.23 |  | 0.1 |  | -1.04 |  | 0.2 |  | 0.9 |  | < .001 |  | 2 |  | 3 |  | 4 |  |
| Vviq 19 |  | 3.74 |  | 1.25 |  | -0.67 |  | 0.1 |  | -0.64 |  | 0.2 |  | 0.85 |  | < .001 |  | 3 |  | 4 |  | 5 |  |
| Vviq 20 |  | 3.78 |  | 1.21 |  | -0.78 |  | 0.1 |  | -0.36 |  | 0.2 |  | 0.85 |  | < .001 |  | 3 |  | 4 |  | 5 |  |
| Vviq 21 |  | 4 |  | 1.14 |  | -0.99 |  | 0.1 |  | 0.11 |  | 0.2 |  | 0.81 |  | < .001 |  | 3 |  | 4 |  | 5 |  |
| Vviq 22 |  | 3.91 |  | 1.13 |  | -0.92 |  | 0.1 |  | 0.11 |  | 0.2 |  | 0.83 |  | < .001 |  | 3 |  | 4 |  | 5 |  |
| Vviq 23 |  | 3.36 |  | 1.29 |  | -0.31 |  | 0.1 |  | -1.01 |  | 0.2 |  | 0.89 |  | < .001 |  | 2 |  | 4 |  | 4 |  |
| Vviq 24 |  | 3.29 |  | 1.4 |  | -0.27 |  | 0.1 |  | -1.21 |  | 0.2 |  | 0.88 |  | < .001 |  | 2 |  | 3 |  | 5 |  |
| Vviq 25 |  | 3.82 |  | 1.2 |  | -0.77 |  | 0.1 |  | -0.42 |  | 0.2 |  | 0.84 |  | < .001 |  | 3 |  | 4 |  | 5 |  |
| Vviq 26 |  | 3.68 |  | 1.15 |  | -0.59 |  | 0.1 |  | -0.4 |  | 0.2 |  | 0.88 |  | < .001 |  | 3 |  | 4 |  | 5 |  |
| Vviq 27 |  | 3.7 |  | 1.2 |  | -0.62 |  | 0.1 |  | -0.61 |  | 0.2 |  | 0.86 |  | < .001 |  | 3 |  | 4 |  | 5 |  |
| Vviq 28 |  | 3.98 |  | 1.15 |  | -1 |  | 0.1 |  | 0.17 |  | 0.2 |  | 0.81 |  | < .001 |  | 3 |  | 4 |  | 5 |  |
| Vviq 29 |  | 3.92 |  | 1.08 |  | -0.83 |  | 0.1 |  | 0 |  | 0.2 |  | 0.84 |  | < .001 |  | 3 |  | 4 |  | 5 |  |
| Vviq 30 |  | 3.83 |  | 1.09 |  | -0.67 |  | 0.1 |  | -0.33 |  | 0.2 |  | 0.86 |  | < .001 |  | 3 |  | 4 |  | 5 |  |
| Vviq 31 |  | 3.89 |  | 1.11 |  | -0.77 |  | 0.1 |  | -0.17 |  | 0.2 |  | 0.84 |  | < .001 |  | 3 |  | 4 |  | 5 |  |
| Vviq 32 |  | 3.73 |  | 1.2 |  | -0.69 |  | 0.1 |  | -0.45 |  | 0.2 |  | 0.86 |  | < .001 |  | 3 |  | 4 |  | 5 |  |
|  | | | | | | | | | | | | | | | | | | | | | | | |

Note. VVIQ = Vividness of Visual Imagery Questionnaire.

**The following results are extracted directly from the R code using the” lavaan” package:**

**1) Unidimensional Model code:**

model <-'

V =~ vviq1 + vviq2 + vviq3 + vviq4 + vviq5 + vviq6 + vviq7 +

vviq8 + vviq9 + vviq10 + vviq11 + vviq12 + vviq13 +

vviq14 + vviq15 + vviq16 + vviq17 + vviq18 + vviq19 +

vviq20 + vviq21 + vviq22 + vviq23 + vviq24 + vviq25 +

vviq26 + vviq27 + vviq28 + vviq29 + vviq30 + vviq31 +

vviq32

'

**2) Bifactor Model Code:**

model <-'

# measurement model

G =~ 1*vviq1 + vviq2 + vviq3 + vviq4 + vviq5 + vviq6 +

vviq7 + vviq8 + vviq9 + vviq10 + vviq11 + vviq12 +

vviq13 + vviq14 + vviq15 + vviq16 + vviq17 + vviq18 +

vviq19 + vviq20 + vviq21 + vviq22 + vviq23 + vviq24 +

vviq25 + vviq26 + vviq27 + vviq28 + vviq29 + vviq30 +

vviq31 + vviq32

S1 =~ 1*vviq1 + vviq2 + vviq3 + vviq4

S2 =~ 1*vviq5 + vviq6 + vviq7 + vviq8

S3 =~ 1*vviq9 + vviq10 + vviq11 + vviq12

S4 =~ 1*vviq13 + vviq14 + vviq15 + vviq16

S5 =~ 1*vviq17 + vviq18 + vviq19 + vviq20

S6 =~ 1*vviq21 + vviq22 + vviq23 + vviq24

S7 =~ 1*vviq25 + vviq26 + vviq27 + vviq28

S8 =~ 1*vviq29 + vviq30 + vviq31 + vviq32

# (residual) (co)variances

S1 ~~ 1*S1

S2 ~~ 1*S2

S3 ~~ 1*S3

S4 ~~ 1*S4

S5 ~~ 1*S5

S6 ~~ 1*S6

S7 ~~ 1*S7

S8 ~~ 1*S8'

**3) Unidimensional + Correlated Residuals Model (Final Model) code:**

model <-'

V =~ vviq1 + vviq2 + vviq3 + vviq4 + vviq5 + vviq6 + vviq7 +

vviq8 + vviq9 + vviq10 + vviq11 + vviq12 + vviq13 +

vviq14 + vviq15 + vviq16 + vviq17 + vviq18 + vviq19 +

vviq20 + vviq21 + vviq22 + vviq23 + vviq24 + vviq25 +

vviq26 + vviq27 + vviq28 + vviq29 + vviq30 + vviq31 +

vviq32

# (residual) (co)variances

vviq1 ~~ vviq2

vviq1 ~~ vviq3

vviq1 ~~ vviq4

vviq2 ~~ vviq3

vviq2 ~~ vviq4

vviq3 ~~ vviq4

vviq5 ~~ vviq6

vviq5 ~~ vviq7

vviq5 ~~ vviq8

vviq6 ~~ vviq7

vviq6 ~~ vviq8

vviq7 ~~ vviq8

vviq9 ~~ vviq10

vviq9 ~~ vviq11

vviq9 ~~ vviq12

vviq10 ~~ vviq11

vviq10 ~~ vviq12

vviq11 ~~ vviq12

vviq13 ~~ vviq14

vviq13 ~~ vviq15

vviq13 ~~ vviq16

vviq14 ~~ vviq15

vviq14 ~~ vviq16

vviq15 ~~ vviq16

vviq17 ~~ vviq18

vviq17 ~~ vviq19

vviq17 ~~ vviq20

vviq18 ~~ vviq19

vviq18 ~~ vviq20

vviq19 ~~ vviq20

vviq21 ~~ vviq22

vviq21 ~~ vviq23

vviq21 ~~ vviq24

vviq22 ~~ vviq23

vviq22 ~~ vviq24

vviq23 ~~ vviq24

vviq25 ~~ vviq26

vviq25 ~~ vviq27

vviq25 ~~ vviq28

vviq26 ~~ vviq27

vviq26 ~~ vviq28

vviq27 ~~ vviq28

vviq29 ~~ vviq30

vviq29 ~~ vviq31

vviq29 ~~ vviq32

vviq30 ~~ vviq31

vviq30 ~~ vviq32

vviq31 ~~ vviq32'

Supplementary Table 9. Standardized factor loadings for the final CFA model.

|  | **Loading** | **R^2^** | **Std. Error** | **z** | **95% CI**  **[Lower - Upper]** | **Std. Est. (all)** |
| --- | --- | --- | --- | --- | --- | --- |
| V →vviq1 | 0.72 | 0.34 | 0.06 | 11.98 | [0.6 - 0.84] | 0.58 |
| V**→**vviq2 | 0.67 | 0.31 | 0.06 | 11.86 | [0.56 - 0.78] | 0.56 |
| V→vviq3 | 0.58 | 0.25 | 0.05 | 11.28 | [0.48 - 0.68] | 0.5 |
| V**→**vviq4 | 0.62 | 0.28 | 0.06 | 11.16 | [0.51 - 0.73] | 0.53 |
| V→vviq5 | 0.62 | 0.28 | 0.05 | 12.44 | [0.52 - 0.71] | 0.53 |
| V**→**vviq6 | 0.69 | 0.32 | 0.06 | 12.4 | [0.58 - 0.8] | 0.57 |
| V→vviq7 | 0.66 | 0.31 | 0.05 | 12.22 | [0.56 - 0.77] | 0.55 |
| V**→**vviq8 | 0.79 | 0.38 | 0.06 | 13.13 | [0.67 - 0.91] | 0.62 |
| V→vviq9 | 0.81 | 0.4 | 0.06 | 13.51 | [0.69 - 0.93] | 0.63 |
| V**→**vviq10 | 0.62 | 0.28 | 0.05 | 12.05 | [0.52 - 0.72] | 0.53 |
| V**→**vviq11 | 0.61 | 0.27 | 0.05 | 11.74 | [0.51 - 0.71] | 0.52 |
| V**→**vviq12 | 0.83 | 0.41 | 0.06 | 13.12 | [0.7 - 0.95] | 0.64 |
| V**→**vviq13 | 0.75 | 0.36 | 0.06 | 13.43 | [0.64 - 0.86] | 0.6 |
| V**→**vviq14 | 0.99 | 0.49 | 0.07 | 14.91 | [0.86 - 1.12] | 0.7 |
| V**→**vviq15 | 0.88 | 0.43 | 0.06 | 14.36 | [0.76 - 1] | 0.66 |
| V**→**vviq16 | 1.18 | 0.58 | 0.08 | 15.55 | [1.03 - 1.33] | 0.76 |
| V**→**vviq17 | 0.8 | 0.39 | 0.06 | 13.53 | [0.68 - 0.92] | 0.62 |
| V**→**vviq18 | 0.75 | 0.36 | 0.05 | 13.85 | [0.64 - 0.86] | 0.6 |
| V**→**vviq19 | 1.06 | 0.53 | 0.07 | 15.36 | [0.93 - 1.2] | 0.73 |
| V**→**vviq20 | 1.02 | 0.51 | 0.06 | 15.86 | [0.9 - 1.15] | 0.72 |
| V**→**vviq21 | 0.94 | 0.47 | 0.07 | 14.37 | [0.81 - 1.07] | 0.68 |
| V**→**vviq22 | 1.17 | 0.58 | 0.08 | 15.33 | [1.02 - 1.32] | 0.76 |
| V**→**vviq23 | 0.86 | 0.42 | 0.06 | 14.89 | [0.74 - 0.97] | 0.65 |
| V**→**vviq24 | 0.91 | 0.45 | 0.06 | 15.31 | [0.79 - 1.02] | 0.67 |
| V**→**vviq25 | 0.73 | 0.35 | 0.05 | 14.07 | [0.63 - 0.83] | 0.59 |
| V**→**vviq26 | 0.77 | 0.37 | 0.05 | 14.92 | [0.67 - 0.87] | 0.61 |
| V**→**vviq27 | 0.85 | 0.42 | 0.06 | 15.3 | [0.74 - 0.96] | 0.65 |
| V**→**vviq28 | 0.9 | 0.45 | 0.06 | 14.55 | [0.78 - 1.02] | 0.67 |
| V**→**vviq29 | 0.87 | 0.43 | 0.06 | 14.81 | [0.75 - 0.99] | 0.66 |
| V**→**vviq30 | 0.92 | 0.46 | 0.06 | 14.62 | [0.8 - 1.05] | 0.68 |
| V**→**vviq31 | 0.9 | 0.45 | 0.06 | 14.43 | [0.77 - 1.02] | 0.67 |
| V**→**vviq32 | 1.05 | 0.52 | 0.07 | 15.95 | [0.92 - 1.17] | 0.72 |
|  |  |  |  |  |  |  |

Note. All factor loadings were significant (p< 0.001)

**Appendix**

**Vividness of Visual Imagery Questionnaire – 2 Persian (VVIQ_2Pr)**

**خواهشمند است پیش از پاسخگویی به پرسش‌نامه، توضیحات زیر را به‌دقت مطالعه فرمایید با تشکر**

**«تصویرسازی ذهنی» به توانایی تجسم‌کردن اشاره دارد، یعنی همان توانایی ایجاد تصاویر ذهنی یا «دیدن در ذهن».**

سؤالات این آزمون، احتمالاً تصاویری خاص را در ذهن شما ایجاد کنند. از شما خواسته می‌شود تا وضوح هر تصویر را بر اساس مقیاس پنج‌درجه‌ای که در زیر ارائه شده است، مشخص کنید. به‌عنوان‌مثال، اگر تصویرِ ذهنیِ شما، «مبهم و غیرشفاف» است، امتیاز 2 به آن بدهید.

1. **در طول آزمون، هنگام ارزیابی وضوح تصور خود، به مقیاس پنج‌درجه‌ای (از 1 تا 5) مراجعه کنید. تلاش کنید هر سؤال را به‌طور مستقل و بدون توجه به پاسخ‌های قبلی، پاسخ دهید.**
2. این آزمون شامل ۳۲ سؤال است که باید با **چشمان بسته** تصور شوند.
3. **وقتی از «چشمان بسته» صحبت می‌کنیم، منظور این است که 1) متن توضیح صحنه را بخوانید، 2) چشمانتان را ببندید، 3) تصویر را در ذهن خود با چشمان بسته تشکیل دهید و سپس 4) چشمانتان را باز کنید تا امتیاز هر مورد را بنویسید.**

**لطفاً تا زمانی که سؤالات صفحه‌ای که در حال انجام آن هستید کامل نشده است، به صفحه بعدی نروید و به سؤالاتی که قبلاً انجام داده‌اید نگاه نکنید. تلاش کنید هر مورد را جداگانه و بدون توجه به امتیاز سایر سؤالات ارزیابی کنید.**

- **مبنای امتیازدهی:** تجسم (تصویرسازی) هر سؤال می‌تواند این‌گونه باشد.

| نوع تجربۀ ذهنی ایجاد شده | امتیاز |
| --- | --- |
| کاملاً واضح؛ در حدِ وضوح و روشنی دیدن طبیعی | 5 |
| نسبتاً واضح و شفاف | 4 |
| تا حدی واضح و شفاف | 3 |
| مبهم و تار (شفافیت کم) | 2 |
| هیچ تصویر ذهنی به وجود نمی‌آید، فقط «می‌دانید» که دارید به آن چیز فکر می‌کنید. | 1 |

- **شروع آزمون:**

| عبارت موارد 1 تا 4:  به یکی از اقوام یا دوستانی فکر کنید که اغلب او را می‌بینید (اما در حال حاضر با شما نیست) و تصویری را که در ذهن شما شکل می‌گیرد بادقت در نظر بگیرید. | | | | | | | |
| --- | --- | --- | --- | --- | --- | --- | --- |
| 1 | 1 | **شکل دقیق صورت، سر، شانه‌ها و بدن.** | 1 | 2 | 3 | 4 | 5 |
| 2 | 2 | **حالت‌های سر، طرز قرارگیری بدن و غیره** | 1 | 2 | 3 | 4 | 5 |
| 3 | 3 | **طرز دقیق راه‌رفتن، طول گام‌ها و غیره** | 1 | 2 | 3 | 4 | 5 |
| 4 | 4 | **رنگ‌های مختلف بعضی از لباس‌هایی که به طور معمول می‌پوشد.** | 1 | 2 | 3 | 4 | 5 |
| عبارت موارد 5 تا 8:  به طلوع خورشید فکر کنید. تصویری را که در ذهن شما شکل می‌گیرد بادقت در نظر بگیرید. | | | | | | | |
| 5 | 5 | **خورشید در حال طلوع از افق، از بالای یک آسمان مه‌آلود است.** | 1 | 2 | 3 | 4 | 5 |
| 6 | 6 | **آسمان صاف می‌شود و خورشید را با رنگ آبی در بر می‌گیرد.** | 1 | 2 | 3 | 4 | 5 |
| 7 | 7 | **ابرها. طوفانی همراه با رعدوبرق شروع می‌شود.** | 1 | 2 | 3 | 4 | 5 |
| 8 | 8 | **رنگین‌کمان ظاهر می‌شود.** | 1 | 2 | 3 | 4 | 5 |
| عبارت موارد 9 تا 12:  به نمای جلوی مغازه‌ای که اغلب به آن می‌روید، فکر کنید. تصویری را که در ذهن شما ایجاد می‌شود در نظر بگیرید. | | | | | | | |
| 9 | 9 | **نمای ظاهری مغازه، از سمت دیگر خیابان.** | 1 | 2 | 3 | 4 | 5 |
| 10 | 10 | **ویترین مغازه، شامل رنگ، شکل و جزئیات کالاهایی که برای فروش هستند.** | 1 | 2 | 3 | 4 | 5 |
| 11 | 11 | **شما نزدیک ورودی مغازه هستید. رنگ، شکل و جزئیات درب مغازه.** | 1 | 2 | 3 | 4 | 5 |
| 12 | 12 | **وارد مغازه می‌شوید و به سمت پیشخوان می‌روید. متصدی سفارش شما را انجام می‌دهد. پول بین شمارد و بدل می‌شود.** | 1 | 2 | 3 | 4 | 5 |
| عبارت موارد 13 تا 16:  به یک منظرۀ روستایی فکر کنید که در آن درختان، کوه‌ها و دریاچه وجود دارد. تصویری را که در ذهن شما شکل می‌گیرد در نظر بگیرید. | | | | | | | |
| 13 | 13 | **خطوط کلی منظره.** | 1 | 2 | 3 | 4 | 5 |
| 14 | 14 | **رنگ و شکل درختان.** | 1 | 2 | 3 | 4 | 5 |
| 15 | 15 | **رنگ و شکل دریاچه.** | 1 | 2 | 3 | 4 | 5 |
| 16 | 16 | **باد شدیدی به سمت درخت‌ها و دریاچه می‌وزد و موج ایجاد می‌کند.** | 1 | 2 | 3 | 4 | 5 |
| عبارت موارد 17 تا 20:  تصور کنید که یکی از اقوام یا دوستانتان شما را با ماشینی پرسرعت در یک بزرگراه می‌برد. تصویری را که در ذهنتان شکل می‌گیرد در نظر بگیرید. | | | | | | | |
| 17 | 17 | **تعداد زیادی ماشین می‌بینید که با حداکثر سرعت در اطراف ماشین شما حرکت می‌کنند. ظاهر کلی ماشین‌ها، رنگ، اندازه و شکل آنها.** | 1 | 2 | 3 | 4 | 5 |
| 18 | 18 | **ماشین شما ناگهان شتاب می‌گیرد تا از ماشین‌های جلو سبقت بگیرد. هنگام عبور، حالت اضطراب را در چهرۀ راننده و افراد داخل خودروهای دیگر می‌بینید.** | 1 | 2 | 3 | 4 | 5 |
| 19 | 19 | **یک کامیون بزرگ از پشت سر چراغ می‌زند. ماشین شما سریع کنار می‌رود تا به کامیون اجازه عبور بدهد. رانندۀ کامیون دست تکان می‌دهد و از شما تشکر می‌کند.** | 1 | 2 | 3 | 4 | 5 |
| 20 | 20 | **شما یک خودروی خراب را کنار جاده می‌بینید. چراغ‌های راهنمایش در حال چشمک‌زدن هستند. راننده نگران است و در حال تماس با تلفن همراه است.** | 1 | 2 | 3 | 4 | 5 |
| عبارت موارد 21 تا 24:  به ساحل کنار اقیانوس، در یک روز گرم تابستانی فکر کنید. تصویری را که در ذهن شما شکل می‌گیرد در نظر بگیرید. | | | | | | | |
| 21 | 21 | **ظاهر کلی و رنگ آب، موج‌ها و آسمان.** | 1 | 2 | 3 | 4 | 5 |
| 22 | 22 | **مردم در حال شنا و آب‌بازی هستند. برخی با یک توپ رنگارنگ ساحلی بازی می‌کنند.** | 1 | 2 | 3 | 4 | 5 |
| 23 | 23 | **یک کشتی اقیانوس‌پیما از افق عبور می‌کند. ردی از دود در آسمان آبی به جا می‌گذارد.** | 1 | 2 | 3 | 4 | 5 |
| 24 | 24 | **یک بالن هوایی زیبا با چهار نفر سرنشین ظاهر می‌شود. بالن از کنار شما عبور می‌کند و تقریباً مستقیم از بالای سرتان عبور می‌کند. سرنشینان دست تکان می‌دهند و لبخند می‌زنند. شما هم به آن‌ها دست تکان می‌دهید و لبخند می‌زنید.** | 1 | 2 | 3 | 4 | 5 |
| عبارت موارد 25 تا 28:  به یک ایستگاه قطار یا مترو فکر کنید. تصویری را که در ذهن شما شکل می‌گیرد، در نظر بگیرید. | | | | | | | |
| 25 | 25 | **نمای کلی ایستگاه که روبروی ورودی اصلی دیده می‌شود.** | 1 | 2 | 3 | 4 | 5 |
| 26 | 26 | **وارد ایستگاه می‌شوید. رنگ، شکل و جزئیات سالن ورودی.** | 1 | 2 | 3 | 4 | 5 |
| 27 | 27 | **به سمت باجۀ فروش بلیت می‌روید، به پیشخوان خالی نزدیک می‌شوید و بلیت خود را می‌خرید.** | 1 | 2 | 3 | 4 | 5 |
| 28 | 28 | **به سمت سکو می‌روید و سایر مسافران و خط راه‌آهن را می‌بینید. قطاری می‌رسد. سوار قطار می‌شوید.** | 1 | 2 | 3 | 4 | 5 |
| عبارت موارد 29 تا 32:  در نهایت به باغی با چمن، بوته، گل و درختچه‌ها فکر کنید. تصویری را که در ذهن شما شکل می‌گیرد، در نظر بگیرید. | | | | | | | |
| 29 | 29 | **نمای کلی و طراحی باغ.** | 1 | 2 | 3 | 4 | 5 |
| 30 | 30 | **رنگ و شکل بوته‌ها و درختچه‌ها.** | 1 | 2 | 3 | 4 | 5 |
| 31 | 31 | **رنگ و ظاهر گل‌ها.** | 1 | 2 | 3 | 4 | 5 |
| 32 | 32 | **چند پرنده روی چمن فرود می‌آیند و شروع به نوک زدن، برای پیداکردن غذا می‌کنند.** | 1 | 2 | 3 | 4 | 5 |
